# Quantification of Lansoprazole in Oral Suspension by Ultra-High-Performance Liquid Chromatography Hybrid Ion-Trap Time-of-Flight Mass Spectrometry

**DOI:** 10.1155/2011/832414

**Published:** 2011-06-22

**Authors:** Stacy D. Brown, Justin D. Connor, Nicholas C. Smallwood, Ralph A. Lugo

**Affiliations:** ^1^Department of Pharmaceutical Sciences, Bill Gatton College of Pharmacy East Tennessee State University, Box 70594, Johnson City, TN 37614, USA; ^2^Department of Pharmacy Practice, Bill Gatton College of Pharmacy East Tennessee State University, Box 70594, Johnson City, TN 37614, USA

## Abstract

An LC-MS/MS method was developed and validated to be used as a stability indicating assay for the study of a 3 mg/mL lansoprazole oral suspension. The method utilizes a UPLC (ultra-performance liquid chromatography) column and unique mass spectrometric detection (ion-trap time-of-flight (IT-TOF)) to achieve a sensitive (LOD 2 ng/mL), accurate, and reproducible quantification of lansoprazole. This method reports an intraday and interday coefficient of variation of 2.98 ± 2.17% (n = 5 for each concentration for each day) and 3.07 ± 0.89% (n = 20 for each concentration), respectively. Calibration curves (5–25 **μ**g/mL) were found to be linear with an R^2^ value ranging from 0.9972 to 0.9991 on 4 different days. Accuracy of the assay, expressed as % error, ranged from 0.30 to 5.22%. This method is useful for monitoring the stability of lansoprazole in oral suspension.

## 1. Introduction

A significant problem in pediatric pharmacotherapy is the lack of commercially available liquid formulations. Proton pump inhibitors (PPIs) are a class of drugs that are routinely used in children; however, stability data for extemporaneous liquid formulations are not readily available for many of these compounds. In a prospective study performed by Lugo et al., which included 21 children's hospitals, an oral suspension of lansoprazole was the number one reported extemporaneous formulation prepared in the inpatient setting [1]. In the study, 19 of 21 hospitals surveyed reported using a 3 mg/mL lansoprazole oral suspension; however, there are limited data regarding the stability of this formulation [1]. Compromised stability, in regard to commercial and extemporaneous formulations, is defined as loss of more than 10% of the active ingredient [2]. While the lack of stability of lansoprazole in acidic media (such as apple juice) has been clearly demonstrated [3], the studies examining the stability in a basic suspension (prepared in 8.4% sodium bicarbonate) are conflicting. According to a study by DiGiacinto et al., the reported stability of lansoprazole suspension was eight hours at 22°C and 14 days at 4°C [4]. In contrast, a study performed by Phillips et al. revealed a stability of 4 weeks when lansoprazole was stored in amber plastic vials under refrigeration and 2 weeks at room temperature [5]. Thus, the commonly accepted stability of lansoprazole suspension prepared in 8.4% sodium bicarbonate is 14 days [6]. 

A few reports of lansoprazole quantification by LC-MS/MS can be found in the literature. Hishinuma et al. [7] measured lansoprazole and rabeprazole in human serum for applications in pharmacokinetic studies using a triplequadrupole mass spectrometer. Oliveira et al. [8] and Wu et al. [9] used similar instrumentation for quantification of lansoprazole for bioequivalence studies. Other reported quantitative methods for this drug rely on UV-VIS spectroscopy [10–12]. The only published method claiming stability-indicating properties for lansoprazole utilizes thin-layer chromatography and UV-VIS spectroscopy [13].

The hybrid ion-trap time-of-flight mass spectrometer configuration is relatively new and was released to the market as a competitor for the Orbitrap (Thermo Scientific, Waltham, MAss, USA) for applications in proteomics. To date, this MS configuration has found applications in the proteomics field [14], as well as in metabolomics and global metabolite profiling [15] and lipidomics [16]. One quantitative method involving the MS configuration has been published measuring five lignan standards applicable to herbal medicines [17]. These investigators noted similar linearity, precision, accuracy compared to the well-established quantitative powers of the triplequadrupole MS configuration; however, the IT-TOF performed at higher sensitivity for all compounds measured [17].

## 2. Materials and Methods

### 2.1. Equipment and Materials

The lansoprazole and omeprazole standards were of USP grade and were purchased from Spectrum Chemical (Gardena, Calif, USA). The solvents used included methanol, water, and 0.1% v/v formic acid in acetonitrile. All of these solvents were of LC-MS grade (Burdick & Jackson, Morristown, NJ, USA). Lansoprazole delayed-release capsules, USP, of 30 mg were used in the pilot stability study (TEVA Pharmaceuticals USA, Sellersville, Pa, USA). The suspension samples were prepared using USP Grade 8.4% w/v sodium bicarbonate (Hospira, Inc., Lake Forest, Il, USA). The HPLC column was a Waters Acquity UPLC BEH C18 column, 1.7 micron, 2.1 × 100 mm (Milford, Ma, USA). The Shimazdu liquid chromatography system consisted of two LC-20AD pumps with UFLC-XR upgrade, SIL-20ACHT autosampler, CTO-20A column oven, DGU-20A_3_ degasser, and CBM-20A Communications module. This system was coupled to the Shimazdu IT-TOF mass spectrometer with an electrospray (ESI) source (Columbia, Md, USA).

### 2.2. LC-MS/MS Conditions

All chromatographic separations were performed using the Waters Acquity UPLC BEH C18 (1.7 micron, 2.1 × 100 mm) column. The isocratic separation utilized a mobile phase of 60% water/40% acetonitrile with 0.1% v/v formic acid at a flow rate of 0.200 mL/min. All injections used a volume of 1 *μ*L. All mass spectrometric measurements were performed using the Shimazdu IT-TOF with an ESI source operating in positive ion mode. The detector voltage was set at 1.45 kV. Both the source temperature and CDL were kept at 200°C. Liquid nitrogen was used as the nebulizing gas at a flow rate of 1.5 L/min. For quantification of lansoprazole and omeprazole, a direct MS/MS method was used, where the transitions specific to the analyte and internal standard were monitored (m/z 370 → 252 and m/z 346 → 198 for lansoprazole and omeprazole, resp.). A 10 msec ion acquisition time was used for each MS2 channel.

### 2.3. LC-MS/MS Validation Experiments

Calibration and validation standards were prepared in 50/50 v/v water/methanol mixture. The calibration curve consisted of five points 5, 10, 15, 20, and 25 *μ*g/mL lansoprazole. These calibration standards were prepared using a 100 *μ*g/mL stock solution of lansoprazole in 50/50 water/methanol. Each calibration and validation solution contained 10 *μ*g/mL omeprazole. All calibration and validation samples were filtered using a 0.22-micron syringe filter prior to injection. Quantification was performed using the peak area ratios between lansoprazole and omeprazole. Five replicates of each of the calibration points were prepared on each day of validation to assess precision and accuracy. Precision was calculated as the relative standard deviation, % RSD = 100 ∗ (SD/mean, SD standard deviation). To reflect the accuracy of the assay, the % error was calculated as the percent difference between the theoretical concentrations and the experimentally determined concentrations of the replicate samples. The validation experiments were repeated over a period of 4 days. The method limit of detection (LOD) was determined using a 3 : 1 signal-to-noise criterion.

### 2.4. Pilot Stability Study Experiment

A lansoprazole suspension (3 mg/mL) was prepared by pouring the contents of ten 30 mg capsules of lansoprazole into 100 mL of 8.4% sodium bicarbonate and stirring on a magnetic stir plate for a minimum of 30 minutes. The sample was stored at room temperature (22°C) and sampled at the following time points: 0 hr, 8 hr, 24 hr, 48 hr, 72 hr, and 168 hr. Upon sampling, 0.600 mL of suspension was removed by micropipette and added to 8.4 mL of a 50/50 mix of methanol and water and vortex mixed. From this mixture, 100 *μ*L was removed by micropipette and added to 800 *μ*L of the 50/50 solvent mix. A volume of 100 *μ*L of the internal standard stock solution (100 *μ*g/mL), omeprazole, was added to the aforementioned mixture for a final concentration of 10 *μ*g/mL omeprazole. This dilution of lansoprazole used was intended to bring the final sample concentration within the calibration range (5–25 *μ*g/mL). The actual concentration of lansoprazole in each sample was calculated using the calibration curve from that day. Before being added to the autosampler vials, suspension samples were filtered using a 0.22-micron syringe filter.

## 3. Results and Discussion

### 3.1. Method Validation

The method was validated in a range of 5–25 *μ*g/mL. Using a 3 : 1 signal-to-noise ratio, the limit of detection (LOD) was determined to be 2 ng/mL (0.002 ng on-column). The actual limit of quantification (LOQ) is likely lower than the lowest validation point, as defined by a 10 : 1 signal-to-noise ratio; however, 5 *μ*g/mL was the lowest concentration validated. It is highly likely that the assay could have been validated in a more sensitive range, but the objective was to create a method that was easily compatible with the 3 mg/mL starting concentration of the lansoprazole suspension. Since the suspension is a nonhomogeneous matrix, the authors felt it best to minimize the number of dilutions needed for sample preparation as well as maximize the volume of suspension used for the samples. Working in the 5–25 *μ*g/mL range allowed for this, thus helping reduce some of the error inherent in sampling from a nonhomogeneous preparation.

Table 1 shows the intraday precision (% RSD) and accuracy (% error) for the method (*n* = 5 for each concentration for each day). The intraday % RSD ranged from 1.28 to 10.54% and the intraday % error from 0.30 to 5.22%.  Table 2 shows the interday precision over four days (% RSD) and accuracy (% error) for the method (*n* = 20 for each concentration). These data indicate a high reliability and reproducibility with an interday % RSD ranging from 1.99 to 3.93% and interday % error from 1.06 to 2.81%. These criteria fall well within what is considered acceptable for method validation [18].

**Figure 1:**
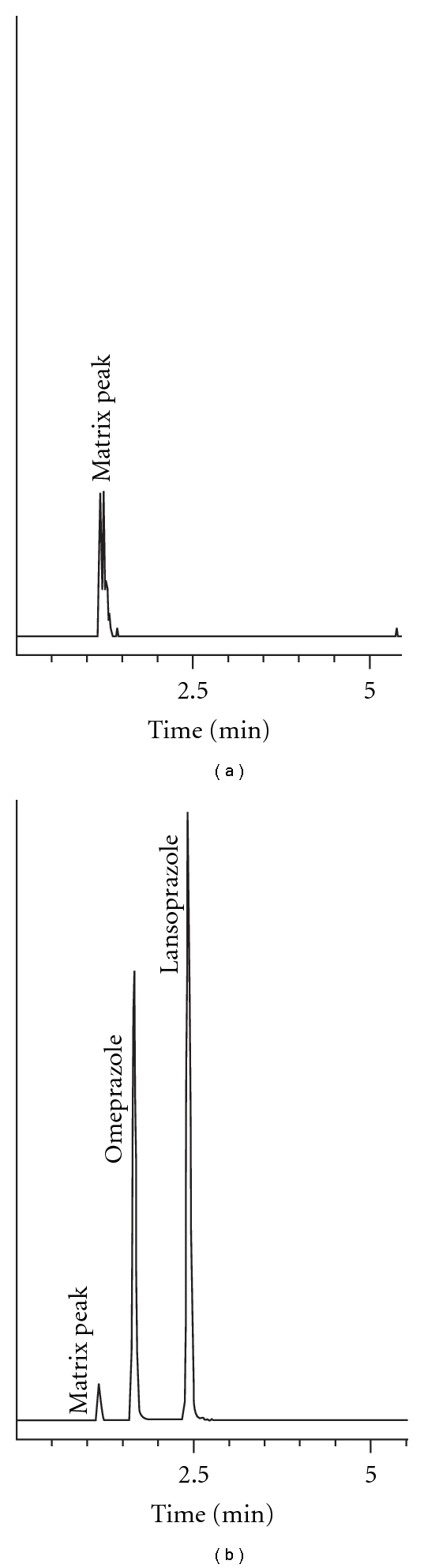
Sample chromatograms showing (a) solvent blank and (b) suspension sample (0 hr).

**Figure 2:**
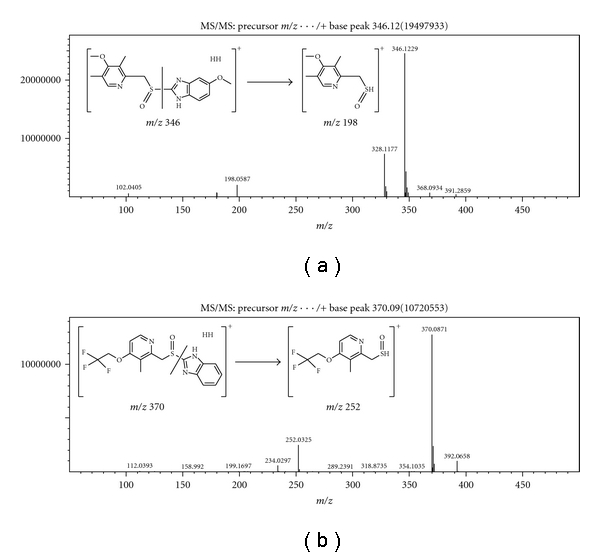
LC-MS/MS transitions monitored for the quantification of (a) omeprazole and (b) lansoprazole.

The use of the Waters Acquity column enabled a separation that was fast (<3 min) and baseline resolved as shown in Figure 1. The matrix peak shown likely represents the presence of phthalate contamination, which has been shown to be ubiquitous in laboratory environments [19]; however, as shown in Figure 1, it is chromatographically resolved from the analyte and internal standard peaks. The direct MS/MS transitions monitored allowed for specificity in quantification of the analyte and internal standard (Figure 2).

### 3.2. Method Application

**Figure 3:**
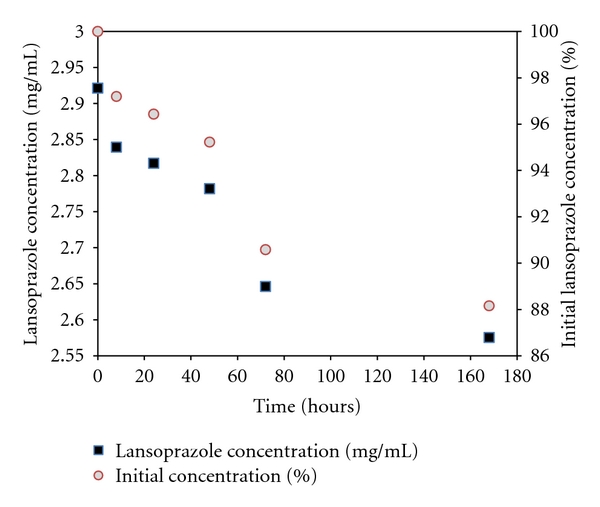
Graphical representation of the results of the pilot stability sample, lansoprazole 3 mg/mL suspension stored at room temperature for 7 days.

To demonstrate that the method was capable of quantifying lansoprazole in a pharmaceutical suspension, a one-sample “pilot study” was run using a 3 mg/mL lansoprazole suspension stored at room temperature. The suspension was sampled periodically and concentration of lansoprazole quantified using the developed LC-MS/MS method. Suspension samples were spiked with internal standard (omeprazole) and diluted to fall within the calibration range as planned for a scaled-up version of the study. The pilot data, shown in Figure 3, indicate that stability of lansoprazole in an oral suspension, if stored at room temperature of 22°C, would be compromised after 72 hours. Loss of stability was defined as lansoprazole concentration <90% of the initial concentration at any time point [2].

## 4. Conclusions

The method presented here is the first application reported of UPLC coupled with the unique IT-TOF mass spectrometric detector to quantify this drug. The use of UPLC is becoming widely accepted as a means to achieve higher-resolution separations compared to conventional HPLC, ultimately resulting in higher sensitivity and fast run times [20]. Furthermore, the use of the IT-TOF mass spectrometer provides the potential for accurate mass data collection on degradation products, something that is not readily achieved using more conventional triple quadrupole instruments. While this method offers a comparable option to existing LC-MS assays for quantification of lansoprazole in terms of accuracy and precision, it stands as one of the few quantitative applications of the hybrid IT-TOF mass analyzer configuration as well as one of two stability-indicating methods for this drug. Ultimately, this method can be applied to monitor the stability of lansoprazole in oral suspensions with confidence of accuracy, precision, and specificity.

## Figures and Tables

**Table 1:** Intraday precision (% relative standard deviation (% RSD)) and accuracy (% error) of the LC-MS/MS assay for quantification of lansoprazole (*n* = 5 for each concentration for each day).

Concentration of lansoprazole added (*μ*g/mL)	Concentration of lansoprazole found (*μ*g/mL)	% Relative standard deviation	% Error
*Day 1*			
* 5*	5.17 ± 0.15	2.90	2.81
* 10*	10.28 ± 0.12	1.16	2.81
* 15*	15.04 ± 0.65	4.35	0.30
* 20*	20.83 ± 0.27	1.28	4.17
* 25*	24.76 ± 0.72	2.91	0.96

* Day 2*			
* 5*	5.12 ± 0.54	10.54	2.06
* 10*	10.31 ± 0.18	1.79	3.07
* 15*	15.15 ± 0.63	4.13	1.02
* 20*	21.04 ± 0.46	2.18	5.22
* 25*	25.04 ± 0.73	2.90	0.15

*Day 3*			
* 5*	4.95 ± 0.15	3.03	0.81
* 10*	9.90 ± 0.11	1.16	0.98
* 15*	14.48 ± 0.63	4.34	3.47
* 20*	20.04 ± 0.26	1.28	0.21
* 25*	23.82 ± 0.69	2.90	4.74

*Day 4*			
* 5*	5.04 ± 0.15	2.98	0.65
* 10*	10.05 ± 0.12	1.16	0.46
* 15*	14.69 ± 0.64	4.34	2.08
* 20*	20.33 ± 0.26	1.28	1.64
* 25*	24.15 ± 0.70	2.90	3.38

**Table 2:** Interday precision over four days (% relative standard deviation (% RSD)) and accuracy (% error) of the LC-MS/MS assay for quantification of lansoprazole (*n* = 20 for each concentration).

Concentration of lansoprazole added (*μ*g/mL)	Concentration of lansoprazole found (*μ*g/mL)	% Relative standard deviation	% Error
* 5*	5.07 + 0.18	3.55	1.18
* 10*	10.13 + 0.20	1.99	1.34
* 15*	14.84 + 0.58	3.93	1.06
* 20*	20.56 + 0.46	2.23	2.81
* 25*	24.44 + 0.89	3.63	2.23

## References

[1] Lugo RA, Cash J, Trimby R, Ward R, Spielberg S (2009). A survey of children’s hospitals on the use of extemporaneous liquid formulations in the inpatient setting. *The Journal of Pediatric Pharmacology and Therapeutics*.

[2] (2006). *Remington: The Science and Practice of Pharmacy*.

[3] Olabisi A, Chen J, Garala M (2007). Evaluation of different lansoprazole formulations for nasogastric or orogastric administration. *Hospital Pharmacy*.

[4] DiGiacinto JL, Olsen KM, Bergman KL, Hoie EB (2000). Stability of suspension formulations of lansoprazole and omeprazole stored in amber-colored plastic oral syringes. *Annals of Pharmacotherapy*.

[5] Phillips JO, Metzler M, Olsen KM (1999). The stability of simplified lansoprazole suspension (SLS). *Gastroenterology*.

[6] Trissel L (2005). *Trissel’s Stability of Compounded Formulations*.

[7] Hishinuma T, Suzuki K, Yamaguchi H (2008). Simple quantification of lansoprazole and rabeprazole concentrations in human serum by liquid chromatography/tandem mass spectrometry. *Journal of Chromatography B*.

[8] Oliveira CH, Barrientos-Astigarraga RE, Abib E, Mendes GD, Da Silva DR, De Nucci G (2003). Lansoprazole quantification in human plasma by liquid chromatography-electrospray tandem mass spectrometry. *Journal of Chromatography B*.

[9] Wu GL, Zhou HL, Shentu JZ, He QJ, Yang BO (2008). Determination of lansoprazole in human plasma by rapid resolution liquid chromatography-electrospray tandem mass spectrometry: application to a bioequivalence study on Chinese volunteers. *Journal of Pharmaceutical and Biomedical Analysis*.

[10] Wahbi AAM, Abdel-Razak O, Gazy AA, Mahgoub H, Moneeb MS (2002). Spectrophotometric determination of omeprazole, lansoprazole and pantoprazole in pharmaceutical formulations. *Journal of Pharmaceutical and Biomedical Analysis*.

[11] Miura M, Tada H, Suzuki T (2004). Simultaneous determination of lansoprazole enantiomers and their metabolites in plasma by liquid chromatography with solid-phase extraction. *Journal of Chromatography B*.

[12] Özaltín N (1999). Determination of Lansoprazole in pharmaceutical dosage forms by two different spectroscopic methods. *Journal of Pharmaceutical and Biomedical Analysis*.

[13] El-Sherif ZA, Mohamed AO, El-Bardeicy MG, El-Tarras MF (2005). Stability-indicating methods for the determination of lansoprazole. *Spectroscopy Letters*.

[14] Wu H, Ge J, Yang P-Y, Wang J, Uttamchandani M, Yao SQ (2011). A peptide aldehyde microarray for high-throughput profiling of cellular events. *Journal of the American Chemical Society*.

[15] Theodoridis G, Gika HG, Wilson ID (2008). LC-MS-based methodology for global metabolite profiling in metabonomics/metabolomics. *Trends in Analytical Chemistry*.

[16] Bisogno T, Piscitelli F, Di Marzo V (2009). Lipidomic methodologies applicable to the study of endocannabinoids and related compounds: endocannabinoidomics. *European Journal of Lipid Science and Technology*.

[17] Liang Y, Hao H, Kang AN (2010). Qualitative and quantitative determination of complicated herbal components by liquid chromatography hybrid ion trap time-of-flight mass spectrometry and a relative exposure approach to herbal pharmacokinetics independent of standards. *Journal of Chromatography A*.

[18] Viswanathan CT, Bansal S, Booth B (2007). Workshop/conference report—quantitative bioanalytical methods validation and implementation: best practices for chromatographic and ligand binding assays. *AAPS Journal*.

[19] Ende M, Spiteller G (1982). Contaminants in mass spectrometry. *Mass Spectrometry Reviews*.

[20] Nováková L, Matysová L, Solich P (2006). Advantages of application of UPLC in pharmaceutical analysis. *Talanta*.

